# Perceptions About the Relationship Between HIV, Antiretroviral Therapy (ART), and Fecundity in Sub-Saharan Africa: Findings from a Systematic Scoping Review

**DOI:** 10.1007/s10461-026-05111-8

**Published:** 2026-04-11

**Authors:** Erica Sedlander, Alison M. El Ayadi, Njeri Wairimu, Jordan Ackernecht, Jennifer Velloza, Marcelle I. Cedars, Kenneth Ngure, Monica Gandhi

**Affiliations:** 1 Department of Social and Behavioral Sciences, Institute for Health and Aging, University of California San Francisco, 490 Illinois Street, San Francisco, CA 94158 USA; 2 Department of Obstetrics, Gynecology and Reproductive Sciences, University of California San Francisco, San Francisco, CA USA; 3 Partners in Health Research and Development, Center for Clinical Research, Kenya Medical Research Institute, Nairobi, Kenya; 4 School of Public Health, University of California Berkeley, Berkeley, CA USA; 5 Department of Epidemiology and Biostatistics, University of California San Francisco, San Francisco, CA USA; 6 Department of Obstetrics, Gynecology and Reproductive Sciences, School of Medicine, University of California San Francisco, San Francisco, CA USA; 7 School of Public Health, Jomo Kenyatta University of Agriculture and Technology, Nairobi, Kenya; 8 Division of HIV, Infectious Disease, & Global Medicine, School of Medicine, University of California San Francisco, San Francisco, CA USA

**Keywords:** HIV, Fertility, Fecundity, Sub-Saharan Africa, Scoping systematic review

## Abstract

Advances in antiretroviral therapy (ART) enable women living with HIV to safely conceive and give birth without vertical transmission. However, in sub-Saharan Africa, 1 in 6 people experience infertility and women living with HIV face even higher rates. Research shows that ART and virologic suppression improve fertility/fecundity, yet this benefit is often overlooked in ART adherence education. To map the literature on people’s understandings and beliefs about the relationship between HIV, ART, and fecundity, a systematic scoping review of the English and French literature in sub-Saharan Africa was conducted. After iterative search strategy development and implementation in PubMed, three independent reviewers screened texts, read a subset of full texts, and used a charting table to summarize results and identify emerging themes. Of 1,981 sources identified, only 12 met the inclusion criteria uncovering the following themes: (1) Beliefs that HIV and PrEP cause infertility exist but have not been widely documented; (2) Perceptions that ART improves health before pregnancy motivates women to adhere and provides hope about future pregnancy; (3) Stigma from providers discourage women living with HIV from seeking preconception care; and (4) Lack of awareness among providers and patients about the impact of HIV on pregnancy, highlights the need for provider training. Research on perceptions of HIV, ART, and fecundity in sub-Saharan Africa is sparse. Future research is needed to determine whether addressing this gap in understanding could improve ART adherence and help couples living with HIV build their families.

**Supplementary Information:**

The online version contains supplementary material available at https://doi.org/10.1007/s10461-026-05111-8.

## Background

A 2022 World Health Organization (WHO) meta-analysis reported that in sub-Saharan Africa (SSA), 1 in 6 people experience infertility—the inability to conceive after 12 months of unprotected sex [1]. Women living with HIV face even higher infertility and subfertility (an extended period of unwanted non-conception without confirmed infertility) rates than their HIV-negative counterparts [2–5]. For example, a study in Côte d’Ivoire found that women who tested positive for HIV had a 17% decreased risk of being pregnant meaning longer intervals of unprotected sex without pregnancy (Cox regression, hazard ratio = 0.83). Moreover, a longer interval was observed among symptomatic women, than asymptomatic women [2]. Additionally, a study in South Africa found that compared with HIV-negative women, women living with HIV not on antiretroviral therapy had a 2-fold increased risk of subfertility (weighted and adjusted risk ratio, 2.00). Infertility risk was attenuated in women on antiretroviral therapy but remained elevated, even after ≥ 2 years on antiretroviral therapy (weighted and adjusted risk ratio, 1.63; 95% confidence interval, 0.98–2.69) [3]. Furthermore, a study using Demographic Health Survey data including objective biomarkers of HIV status from eleven African countries found that women living with HIV had a 25% average reduction in fecundity [4]. Moreover, among a subset of women who unsuccessfully tried to become pregnant for three months, women living with HIV had half the odds of subsequently becoming pregnant compared to women without HIV [4]. A 2021 study from South Africa corroborated this finding, reporting that women with high viral loads (a marker for antiretroviral therapy [ART] non-adherence) are more than twice as likely to miscarry than women who are virally suppressed [6].

Several factors contribute to elevated infertility and subfertility risk among women living with HIV, largely related to immune and endocrine system dysfunction. For example, women living with HIV often have increased rates of prolonged anovulation and amenorrhea, and increased risk and severity of other co-infections, such as chlamydia and gonorrhea, which can lead to pelvic inflammatory disease (PID), a leading cause of tubal factor infertility [7–11]. HIV also affects male fertility, but the sperm abnormalities found in HIV patients are poorly understood, since both the virus and treatment (ART) can cause changes [12, 13]. Despite the varied effects, men on ART show improvement in sperm quality after six months on ART [14].

Infertility and HIV combined can create a high burden of distress in general, but especially in SSA, where motherhood is seen as part of the marriage contract and often defines a woman’s worth [15–17]. A 2024 scoping review on the social impacts of infertility and HIV found that both an HIV diagnosis and infertility were stigmatizing experiences for women who wanted children, but the stigma of infertility had a greater negative impact on women than an HIV diagnosis [18]. A 2025 qualitative study among sero-different couples in Uganda found that having more children helped avoid infertility stigma and was prioritized over HIV prevention [19]. Research among women living with HIV in SSA highlights the normative perception that having children makes you a “full person” [20] and infertility in this setting is responsible for significant social, psychosocial and economic costs [21]. Our prior work in Kenya illuminated the family and societal pressure to have children soon after marriage to prove one’s fertility and as part of the spoken or unspoken contract of marriage [17].

Fortunately, advances in ART allow women with HIV, including women in sero-different relationships, to safely conceive and prevent vertical transmission [22, 23]. In 2015, the WHO began recommending universal ART at HIV diagnosis, regardless of CD4 count or other indicators to improve health status and prevent transmission [24]. Before this, pregnant women were automatically ART eligible but otherwise healthy women were not ART-eligible pre-conception. ART suppresses the virus, making it undetectable—and untransmittable (“undetectable = untransmittable”) [22]. Since this shift, studies show that women on ART have higher fertility rates [25]. A 2016 review found that ART improves women’s fecundity after about a year of consistent use [26]. As ART access expands, the fertility gap between HIV-positive and HIV-negative women continues to close [25, 27, 28]. But ART adherence challenges persist; only 78% of people living with HIV in Eastern and Southern Africa are virally suppressed [29]. Factors influencing adherence are complex, intersectional, and likely gender-specific across settings. Some notable barriers include HIV stigma and confidentiality concerns, lack of family and community support, physical, economic, and emotional stress, depression, alcohol or drug use [30, 31]. Given the multi-level barriers, a multi-faceted approach is needed to address adherence but thus far, awareness around HIV, ART, and fertility has not been utilized as a potential ART driver.

To promote ART adherence and help women and couples reach their family building goals, we need a better understanding of how people perceive the link between HIV, ART, and fecundity. To our knowledge, there has been no systematic or scoping review of people’s perceptions of the relationship between HIV, ART, and fertility/fecundity performed to date. This systematic scoping review fills that gap, collating information from across the literature to provide conceptual guidance for ART adherence efforts and, ultimately, to help couples living with HIV reach their family building goals. Without attention to this area of research, we may be missing salient and potentially effective messages to improve ART adherence.

## Methods

Our scoping review methodology is informed by the Arksey and O’Malley [32] and Levac et al. frameworks which outline the required steps to complete a rigorous review [33] keeping in mind updates to the framework like integrating a team-based approach throughout all stages of the scoping review process. This review is in accordance with Preferred Reporting Items for Systematic Reviews and Meta-Analyses (PRISMA-ScR) guidelines [34]. We opted to conduct a systematic scoping review rather than a systematic review due to the wide-ranging nature of our questions and the fact that our findings are intended to identify research gaps and synthesize the literature rather than guide medical or policy decisions or assess effectiveness [35]. Given the absence of previous reviews, a scoping review is an appropriate first step to explore the existing literature on perceptions of HIV, ART, and fertility [36]. We also chose to include qualitative and quantitative papers because we wanted to examine people’s understandings and beliefs about the relationship between HIV, ART, and fecundity. Both methods are appropriate to understand this intersection (e.g., knowledge can uncover understandings which is often depicted using quantitative methods, but social norms and beliefs are often explored using qualitative methods).

### Objectives

The objective of this scoping review was to map the literature on people’s *understandings and beliefs* of the relationship between HIV and fecundity and people’s *understandings and beliefs* about the *effectiveness of ART* in reducing HIV’s adverse impact on fecundity. Additionally, we aimed to uncover gaps in the literature and to document suggestions for areas of intervention to increase awareness around how HIV and ART affect fecundity. To achieve these objectives, our review targets the following primary and secondary research questions:

#### Primary Research Questions

1. What are people’s understandings of the relationship between *HIV and fecundity* in sub-Saharan Africa?
2. What are people’s understandings of the relationship between *ART and fecundity* in sub-Saharan Africa?

#### Secondary Research Questions

1. What are the authors’ recommendations for interventions to raise awareness around how HIV and ART affect a couple’s ability to conceive?
2. What are the research gaps for future research?

### Key Concepts

In this scoping review, we are referring to fecundity as a woman’s ability to biologically conceive [3]. Fertility refers to the number of children that women have, and fertility preferences are the number of children that women want to have. Subfertility refers to an extended period of unwanted non-conception without confirmed infertility (often defined as not having conceived within six months of attempted conception) [4]. Given that they are often used interchangeably, and we are interested in beliefs, norms and perceptions, we used all terms in our search but during screening and full text review, we only included papers that refer to perceptions about HIV or ART and fecundity.

### Data Sources and Management

PubMed was reviewed and all data and articles were managed within Zotero reference manager (Fairfax, Virginia, USA) and Covidence systematic review (Melbourne, Victoria, Australia) software. After our initial search and discussions with HIV and infertility experts and a medical librarian, we decided not to search databases outside of PubMed or incorporate the grey literature. We made this decision because PubMed captures MEDLINE-indexed journals, non-MEDLINE-indexed journals, includes pre-print papers, and books/chapters, includes over 37 million citations and abstracts, and the top ten major HIV/AIDS journals are indexed there. Additionally, after piloting our search strategy which indicated that the relevant literature was sparse and because this systematic scoping review was not intended to inform medical or policy decisions or assess effectiveness, the review team in conjunction with the medical librarian decided not to expand to additional databases as it was unlikely to yield substantial new eligible studies relative to the added screening burden, although this remains unquantified.

### Search Strategy

A medical librarian with training and experience in systematic and scoping reviews assisted in the design of our search strategy using an iterative process that follows an established search method. A three-step search strategy for identifying published studies was used. First, as part of the piloting phase, a preliminary search in PubMed was conducted to identify key articles on our topic and begin the process of term harvesting, described below. From these key articles, text words and MeSH terms from titles, abstracts and author-supplied keywords were extracted and to build a comprehensive list of keywords and controlled vocabulary terms, to inform our search strategy development.

The search strategy was developed in PubMed, using controlled vocabulary (e.g., MeSH, Emtree, thesaurus terms) where databases allow. The medical librarian reviewed multiple iterations during the piloting phase, including the results and final search strategy, and discussed with authors which terms to add or remove to optimize the search for our research questions, using the Peer Review of Electronic Search Strategies guidelines [37]. Third, the reference lists of all included reports and articles were searched to identify any additional eligible studies (*n* = 9 additional studies were included). All these steps (the medical librarian reviewing and revising our search strategy, checking that the initial list of papers was included in our final search, keeping an audit trail of edits to the search string, and ensuring that our search strategy included the initial list of papers) were part of validating our search strategy.

The search strategy combined three main concepts: (1) HIV/ART; (2) fecundity/fertility/infertility/subfertility; and (3) knowledge/attitudes/beliefs/perceptions. Boolean logic was applied by combining similar key words and controlled vocabulary. Table 1 shows our strategy which produced 1,984 results:

**Table 1:** Search strategy

(HIV [tiab] OR “HIV“[Mesh] OR “Human immunodeficiency virus” [tiab] OR “HIV infections” [tiab]) AND (attitudes [tiab] OR attitude [tiab] OR belief [tiab] OR beliefs [tiab] OR knowledge [tiab] OR perception [tiab] OR perceptions [tiab] OR perspectives [tiab] OR understand [tiab] OR understanding [tiab] OR idea [tiab] OR ideas [tiab]) AND (fecundity [tiab] OR fecund [tiab] OR conceive [tiab] OR childbearing [tiab] OR fertility [tiab] OR fertile [tiab] OR subfertile [tiab] OR subfertility [tiab] OR reproductive [tiab] OR pregnancy [tiab] OR pregnant [tiab])
**AND** (“sub-Saharan Africa ” [tiab] OR Africa [tiab] OR Angola [tiab] OR Benin [tiab] OR Botswana [tiab] OR “Burkina Faso” [tiab] OR Burundi [tiab] OR “Cabo Verde” [tiab] OR Cameroon [tiab] OR “Central African Republic” [tiab] OR Chad [tiab] OR Comoros [tiab] OR Congo [tiab] OR “Côte d’Ivoire” [tiab] OR [tiab] OR Djibouti [tiab] OR Equatorial Guinea [tiab] OR Eritrea [tiab] OR Eswatini [tiab] OR Ethiopia [tiab] OR Gabon [tiab] OR Gambia [tiab] OR Ghana [tiab] OR Guinea [tiab] OR “Guinea-Bisub-Saharan Africa” [tiab] OR Kenya [tiab] OR Lesotho [tiab] OR Liberia [tiab] OR Madagascar [tiab] OR Malawi [tiab] OR Mali [tiab] OR Mauritania [tiab] OR Mauritius [tiab] OR Mayotte [tiab] OR Mozambique [tiab] OR Namibia [tiab] OR Niger [tiab] OR Nigeria [tiab] OR Rwanda [tiab] OR “Sao Tome and Principe” [tiab] OR Senegal [tiab] OR Seychelles [tiab] OR “Sierra Leone” [tiab] OR Somalia [tiab] OR “South Africa” [tiab] OR “South Sudan” [tiab] OR Sudan [tiab] OR Togo [tiab] OR Tanzania [tiab] OR Uganda [tiab] OR Zambia [tiab] OR Zimbabwe [tiab]) NOT (“sero-discordant” NOT discordant [tiab])

The search was conducted on November 1 2024, and included articles from 2000 to 2024. Papers starting from the year 2000 were included because beliefs and information spread from person to person so someone living with HIV now may be influenced by what others in their community (e.g., their grandparents, in-laws, friends, etc.) believed about how HIV and ART affect fertility from years ago [38]. Additionally, while the WHO’s recommendation for universal ART began in 2015, implementation of these guidelines varied across countries [39]. Therefore, studies published up to 15 years prior to the shift in WHO ART recommendations were included.

### Inclusion and Exclusion Criteria

Inclusion and exclusion criteria for selection of eligible studies was developed using the population, concept, context elements proposed for scoping reviews by the Joanna Briggs Institute (Table 2) [40]. No limits were placed on study design; we included both quantitative and qualitative methods. We included studies that took place in, or report on findings from SSA, written in English or French.

**Table 2:** Population, concept and context for identification of eligible studies

Criteria	Description
Population	Any perceptions including of people living with HIV, the providers who care for them, or anyone else (e.g., their social networks including community members, friends, family, and anyone in the general population).
Concept	HIV, ART, and fertility.
Study Type	Both quantitative and qualitative research.
Context	Sub-Saharan Africa; articles written in English or French.

## Population, Concept and Context for Identification of Eligible Studies

### Study Selection

**Fig. 1:**
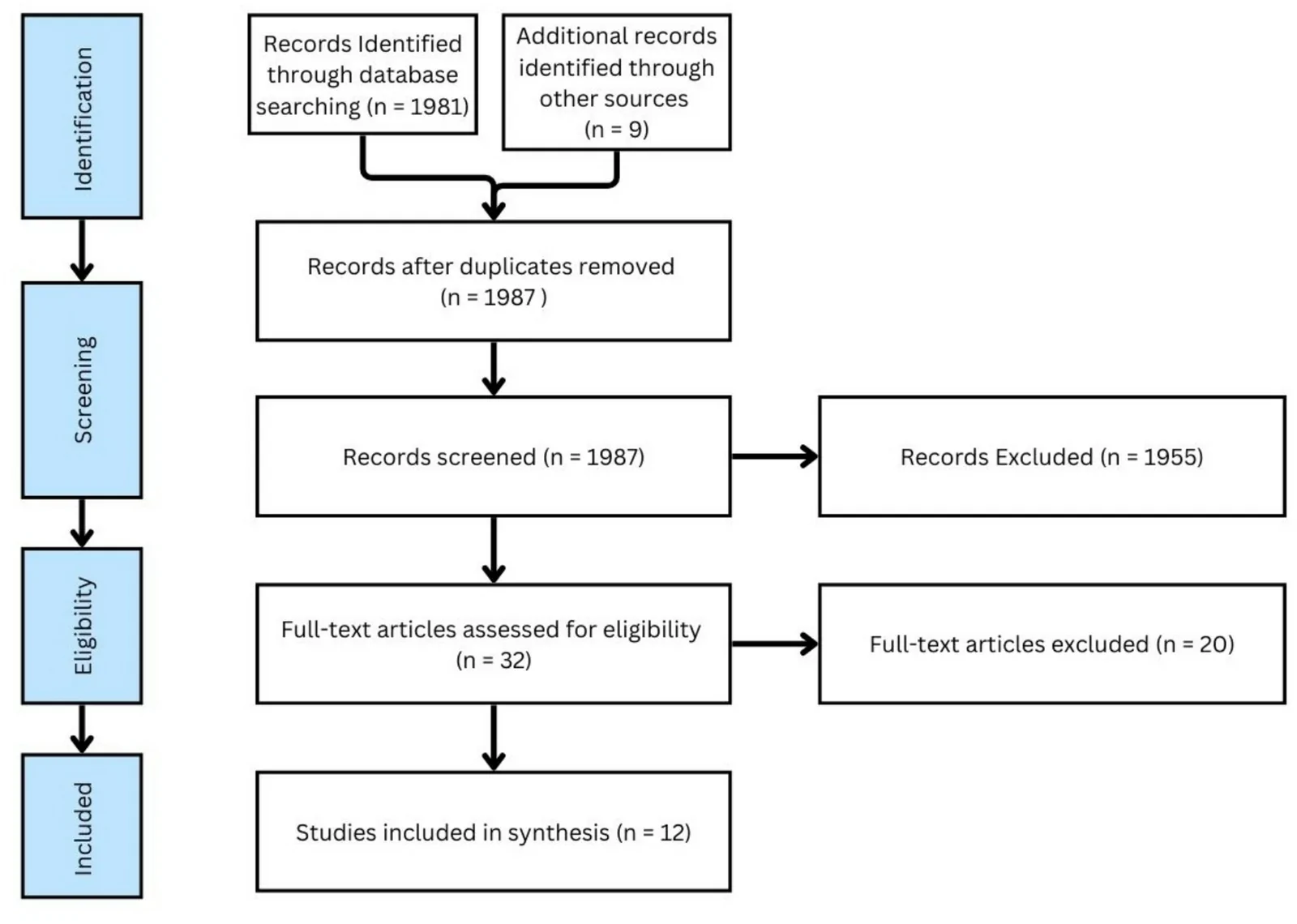
PRISMA flow diagram for study selection process

Three reviewers (co-authors ES, NW, and JA) participated in both steps (1) the screening and (2) the full text review. Two of the reviewers are from U.S. institutions and one reviewer is from a Kenya based institution. Their backgrounds range from public health to anthropology to health services research. Reviewers independently screened titles and abstracts of all articles (two reviewers per article). Reviewers met weekly during the abstract review process to discuss challenges and ambiguities related to study selection. If two reviewers disagreed during the screening process, the third reviewer broke the tie. Study screening progress is documented in a modified PRISMA flow chart (Fig. 1). After the initial search was completed, all citations were imported to Covidence for management and screening. Duplicates were removed and additional records were identified through hand searching including reviewing the references for included papers. In this first step, titles and abstracts were reviewed by two separate reviewers for inclusion. In step (2) full texts were reviewed for definitive classification of inclusion or exclusion. One reviewer was assigned each paper, and another reviewer checked their synthesis and decision. Articles were excluded that ultimately did not align with the inclusion criteria after full text review.

### Extraction of Results

Next, a charting table, created in Excel, was used to record information from each publication. Charting is a method for synthesizing and interpreting qualitative data by sifting, sorting, and analyzing material according to key issues and themes. Table 3 below, “Characteristics and findings of included studies related to perceptions about HIV or ART and fecundity” is the final charting table that reports on results related to our research questions only. All three reviewers (co-authors ES, NW, and JA) participated in this process.

**Table 3:** Characteristics and findings of included studies related to perceptions about HIV or ART and fecundity

Author(s) Year	Country	Data collection methods	Participant Demographics	Findings and Recommendations
King et al. (2011) [41]	Uganda	In-depth interviews	Women living with HIV (*n* = 29), Partners of women living with HIV (*n* = 16) Marital status ·Women: 69% (20/29) married/co-habiting ·Men: 75% (12/16) married/co-habiting Age ·Mean age of women: 37 years ·Mean age of men: 32 years	Couples commonly held the belief that HIV made them infertile. As a result, some did not use family planning (or used inconsistently) so in several cases, pregnancy was a surprise. Both men and women associated ART with positive outcomes such as better physical and emotional health which led to increased sexual desire and ultimately pregnancy for some.
				*Recommendation*: Integrate reproductive health education and family planning services into ART programs for both men and women that focus on restoring fertility following ART initiation.
*Sow (2014) [42]	Senegal	Repeated individual interviews, group interviews, and participant observation (This study combined data from two qualitative investigations)	Healthcare professionals (*n* = 21) Pregnant women (*n* = 20) Women living with HIV(*n* = 25) Marital status ·Women with HIV: all married Age ·Mean age of women with HIV: 32 years	There were concerns about taking ART during pregnancy. Couples sometimes seek guidance from healthcare providers or from women living with HIV who have become mothers. Although all the women surveyed express fear of the negative consequences associated with having multiple pregnancies due to their HIV status, the majority do not use contraception continuously. Social workers seem to be more attentive to the women’s desire to have children than medical doctors. Provider counseling is often limited to the maternal “duty” or “responsibility” to prevent vertical transmission.
				*Recommendations*: In a context where the pressure to bear children is strong, the biomedical system should be re-structured to better support women’s reproductive needs and include men in educational efforts.
Chitukuta et al. (2019) [43]	Malawi Uganda South Africa Zimbabwe	In-depth interviews and focus group discussions	HIV-negative, sexually active women (*n* = 214) Study sites ·South Africa: 98 participants (45.8%) ·Uganda: 39 participants (18.2%) ·Zimbabwe: 39 participants (18.2%) ·Malawi: 38 participants (17.8%) Age ·Mean age: 26 years Marital status ·44.9% were married	Participants believed that the drugs inside the vaginal ring for HIV-1 prevention, were intentionally there to cause infertility so as “to limit the Black population” resulting in fear of using it.
Awiti Ujiji et al. (2010) [44]	Kenya	In-depth interviews	Pregnant women (*n* = 9) Women who had delivered in the past 12 weeks (*n* = 6) Women who were seeking to become pregnant (*n* = 5) Age ·Age range: 22–45 years Marital status ·6 (30%) were single ·6 (30%) were co-habiting ·4 (20%) were married ·4 (20%) were widowed	Some women perceived counselling to improve their CD4 cell count before trying to conceive as restrictive; they felt it could delay pregnancy and were considered tests to determine their capability of getting pregnant.
				*Recommendations*: Counseling should focus on bridging the gap in knowledge between lab test results and health behaviors (like ART adherence), which could help women understand how this may affect their goals for having children.
Nduna and Farlane (2009) [45]	South Africa	Semi-structured interviews, focus groups and a series of participatory workshops	Black women (*n* = 78) Data collection ·15 completed semi-structured interviews ·55 completed the focus group discussions ·8 completed the Stepping Stones workshops Research sites ·Eastern Cape: Four villages and one township in a predominantly rural area ·Gauteng: Multiple townships in a predominantly urban area surrounding Johannesburg Age ·16 years and older Other characteristics ·Participants mainly used public, no-fee or lowest fee charge health services. ·Were of low to middle incomes Mainly resided in townships and villages in either a rural or urban setting. ·Women living with HIV came from clinic-based support groups of people living with HIV.	Women considered their partners desires for children, especially when the partner did not have HIV or other children, which caused them some anxiety. On the one hand, they wanted to meet these desires but were wary of potential health risks. Some participants felt that getting pregnant would be too strenuous on their bodies given their HIV diagnosis. Some expressed a fear of dying because their bodies would be “drained of nutrients” while pregnant and living with HIV. Women felt that ART made them fit for pregnancy as it boosted their immunity but were still worried about whether ART would help them to live long enough to raise their children. They reported that ART helped them fulfill childbearing expectations from partners and families. Communicating fertility desires with healthcare providers was encouraged to receive necessary counselling but women often did not seek preconception counseling/care due to stigma from providers against women living with HIV having children.
				*Recommendations*: Authors noted that women living with HIV need to be informed about how HIV affects pregnancy outcomes and vice versa. Despite healthcare providers willingness to have fertility discussions, they often discourage women living with HIV from getting pregnant.
Saleem et al. (2016) [46]	Tanzania	In-depth interviews	Healthcare providers (*n* = 30), Men living with HIV (*n* = 30) Women living with HIV (*n* = 30) Study sites ·7 purposively selected health facilities providing HIV reproductive and child health, and PMTCT services. ··Health facilities were in two districts; ··Iringa Municipal and Iringa Rural. ··Selected from different levels of the health care system, including the regional hospital, a district hospital, 3 health centers and 2 health dispensaries. ···The health centers and dispensaries were selected to represent both rural and semi-urban settings. Healthcare provider characteristics ·⅓ had a primary affiliation with an HIV clinic, ·½ provided PMTCT services at a reproductive and child health clinic ·3 home-based HIV care providers Men and women living with HIV ·Had received a positive HIV diagnosis ·18–49 years	Healthcare providers felt limited in their capacity to support patients living with HIV about their reproductive health because they lacked training. They reported that they did not know specific policies or updates in national guidelines, so providers often referred these clients to other more qualified providers. Some providers and patients reported that it was important for men and women living with HIV trying to conceive to first get their health checked though providers had different opinions about the recommended CD4 cell count before conception. A few providers discouraged their patients from having children due the effects of pregnancy on their health especially if they already had other children. Similarly, patients were worried that providers would discourage them from having children with some reporting that providers told them they could not have children due to their HIV status.
				*Recommendations*: Integration of HIV and sexual and reproductive health services to support people living with HIV and their partners in planning pregnancies and health management.
Sofolahan and Airhihenbuwa (2013) [47]	South Africa	Focus group discussions	Women living with HIV on ART (*n* = 35 women within four focus group discussions)	Pregnancy promoted adherence to ART as one participant said, *“I normally don’t take pills*,* but I take them for my baby.”* Some women reported that healthcare providers discourage women living with HIV from getting pregnant without proper counseling. *Recommendations*: Capacity-building programs for healthcare workers could ensure that providers are taking into consideration the sexual and reproductive desires of women living with HIV during counselling.
Gutin et al. (2020) [48]	Botswana	In-depth interviews	Sexual and reproductive health/HIV providers (*n* = 10) Women living with HIV who have been pregnant in their lifetime (*n* = 10) Provider characteristics (*n* = 10) Professional roles ·6 (60%) nurses ·3 (30%) midwives ·1 (10%) medical doctor Age ·Mean age: 41 years Women living with HIV (*n* = 10) ·Mean age: 32 years Relationship status ·2 (20%) were single ·3 (30%) were in relationships but not cohabiting ·5 (50%) were cohabiting with a partner or spouse	Healthcare providers had reservations about childbearing including repeat pregnancies, among people living with HIV as they feared vertical transmission, which made women living with HIV feel unsupported and discouraged. Nevertheless, some providers felt that they should offer their clients counselling without preventing them from having children. Others however withheld information about safer conception. *Recommendations*: There is a need to educate healthcare providers about the Undetectable=Untransmittable (U = U) campaign to uphold reproductive rights for women living with HIV and reduce transmission of HIV. Additionally, providers need comprehensive safer conception training to support couples and reduce health risks associated with HIV transmission.
Yeatman (2011) [49]	Malawi	In-depth interviews	Men and women living with HIV (*n* = 48) Interview sample ·23 (47.9%) men ·25 (52.1% women)	Most participants believed that pregnancy would negatively impact their health due to living with HIV. In other words, pregnancy would “weaken their blood” and “make them ill” causing their HIV to progress. The belief that pregnancy is dangerous if you are living with HIV goes hand in hand with its corollary: if you avoid pregnancy, you will live longer.
Laar (2013) [50]	Ghana	Surveys	Heath care workers providing testing and counseling services to HIV patients including nurses (*n* = 32) and medical officers (*n* = 3)	Providers demonstrated a high level of ignorance regarding the various reproductive options available to women living with HIV. Only ~ 10% of the providers were aware of some reproductive options for women living with HIV. A quarter would advise them to have unprotected intercourse as an option to conceive. Some of the providers openly expressed their inability to give qualified and relevant advice to women living with HIV. These findings suggest that patients living with HIV do not receive comprehensive information about their reproductive options.
Kastner et al. (2014) [51]	Uganda	In-depth interviews	Pregnant women receiving ART (*n* = 25) Marital/cohabitation status ·21 (84%) were married/cohabiting Age ·Median age: 29 years	Women reported that counseling on childbearing was largely discouraging, emphasizing the risks of childbirth to their health given their HIV status. Despite perceived dangers of pregnancy while living with HIV, many women noted that social pressures to have children outweighed risks. While formal pregnancy counseling was limited, peer support at clinics provided encouragement, fostering an informal network where women shared experiences. Provider guidance on ART’s role in reducing health risks made women feel hopeful about pregnancy. ART access also increased optimism about childbearing, helping women regain a sense of normalcy, reduce stigma, and balance reproductive roles with their health and family well-being. *Recommendations*: Addressing gaps in clinical counseling is crucial for integrating sexual and reproductive health programs. Supporting healthcare workers to routinely assess fertility goals can improve reproductive care by providing effective contraception for those avoiding pregnancy and safer conception counseling for those who wish to conceive. Integrating HIV and reproductive health services is essential to supporting desired pregnancies and prevent unintended ones. Public health messaging should target women living with HIV, healthcare providers, and communities to raise awareness about pregnancy experiences on ART and ensure informed reproductive choices.
Harries et al. (2007) [52]	South Africa	In-depth interviews	Health care providers (*n* = 14) Public sector policy makers in the HIV field (*n* = 12) 14 (53.8%) health care providers 12 (46.2%) policy makers and managers Health Care Providers (*n* = 14) Age ·Mean age: 38 years Gender ·11 (79%) women ·3 (21%) men Professional roles ·public health doctors ·registered professional nurses ·HIV adherence counselors Policy Makers and Managers (*n* = 12) Age ·Mean age: 48 years Gender ·8 (67%) women ·4 (33%) men Professional background ·Public sector policy makers and managers ·Managers within HIV/AIDS and reproductive health NGOs	Healthcare providers believed that women should have a sufficiently high CD4 count and access to ART before getting pregnant. While others emphasized the importance of reproductive rights and person-centered care. Some doctors were concerned that if healthcare providers overlooked patients’ plans to become pregnant and did not offer proper counseling, those patients might stop their ART treatment. Most policy makers felt that developing counseling guidelines for women who want to get pregnant while living with HIV would be valued. Without guidelines providers would continue “bumbling along” and make “ad hoc” recommendations.

*Published in French

### Data Synthesis

The findings from the scoping review are presented in both a table format with year of the literature, countries of origin, research methods, etc. following the general format of our charting table (Table 3) and in a narrative format. Reviewers thematically analyzed the data according to our two primary and two secondary research questions. Additional content experts (a medical doctor who cares for people living with HIV and a social scientist who conducts HIV and ART adherence research in Kenya) also contributed to interpretation during this step. An excel document included all papers from the full text review (*n* = 32) and described in detail the methods and findings according to each of the four research questions. The findings that were relevant to our research questions were highlighted to determine which to include in the final review and which to discuss whether to include them during meetings. Researchers met weekly during this process to discuss findings within the charting table and ultimately agreed on the twelve papers listed below. Due to the scoping nature of our research questions, the quality of the papers was not appraised.

## Results

Figure 1 shows the PRISMA flow diagram for study selection process. 1981 papers were initially identified. In the first stage, three duplicates were removed. A total of 1955 papers were ineligible and nine were added, which were found through other sources, including a hand search of references. In the second stage, a full text review of 32 papers was conducted. Of the 32 studies, 20 did not meet the inclusion criteria as they were not reporting on our primary or secondary research questions but on tertiary topics such as prevention of vertical transmission, safe conception for sero-different partners (when one partner has HIV and the other does not), and fertility desires (see Supplemental File 1 Excluded Studies). While a total of 12 studies met the study inclusion criteria, the level of relevance to the research questions varied. All included papers referenced the study phenomena but did not report in depth findings. In other words, not a single paper included the aim of reporting on perceptions about the relationship between HIV, ART, and fecundity illuminating a scarcity of literature on this topic.

Table 3 shows information about the selected publications. These papers use data from across sub-Saharan Africa including Uganda, Senegal, Malawi, South Africa, Zimbabwe, Kenya, Tanzania, Botswana, Ghana, and Zambia. Eleven papers were published in English, and one was published in French in a range of journals including public health, reproductive health, and HIV focused journals. The papers were published between 2009 and 2019 but nine out of twelve before 2015 when ART was recommended for all people when diagnosed with HIV regardless of CD4 count. We included eleven qualitative studies and one quantitative study.

This systematic scoping review uncovered four emerging themes about people’s understandings of the relationship between HIV, ART, and fecundity in SSA including: (1) Beliefs that HIV and PrEP cause infertility exist but have not been well documented (two studies); (2) Perceptions that ART improves health before pregnancy motivates women to adhere and provides hope about future pregnancy (five studies); (3) Stigma from providers discourage women living with HIV from seeking preconception care (six studies); and (4) Lack of awareness among both providers and patients about the impact of HIV and ART on pregnancy (eight studies).

### Beliefs that HIV and PrEP Cause Infertility Exist but Have Not Been Widely Documented

There was a limited number of studies reporting that HIV or HIV prevention technologies (e.g., PrEP) cause infertility. Specifically, only two studies directly related to beliefs around HIV and fecundity [41, 43]. One study, conducted in Uganda among women on ART and their partners, reported that couples believed that having HIV would make them infertile, so they were not using contraception [41]. Couples reported that since they were having sex without a condom and not getting pregnant, this was further evidence to corroborate this belief. When some couples subsequently did get pregnant accidentally, they were surprised [41]. There was only one study, that took place in Malawi, Uganda, South Africa and Zimbabwe among HIV negative sexually active women, about HIV prevention (the vaginal ring) and beliefs about infertility. This study uncovered the widespread belief among participants that the vaginal ring for HIV prevention causes infertility. Specifically, they believed that the drugs inside the ring had been put there deliberately to cause infertility and “to limit the Black population” [43]. It is important to note that PrEP beliefs were not originally in the scope of this review but due to the scarcity of literature and the proximity to ART as a medication, we chose to include this study.

Two other studies were related to this topic but did not directly report beliefs around HIV and fecundity. In one study conducted in Senegal among healthcare providers and women living with or without HIV, participants living with HIV reported not using contraception but stated that “they knew they could get pregnant” [42]. Another study conducted in Malawi among men and women living with HIV, reported that women believed that getting pregnant would hasten the progression of their HIV diagnosis making pregnancy too dangerous in general for their health [49], but this study focused more on how pregnancy would negatively affect their HIV prognosis, not their fecundity.

### Perceptions that ART Improves Health Before Pregnancy Motivates Women to Adhere and Provides Hope About Future Pregnancy

Overall, beliefs about ART were positive with women and providers noting the benefits for protecting women’s health, fecundity, and once pregnant, the baby’s health. All studies that reported on ART and fecundity noted positive perceptions around ART and pregnancy including improved physical and emotional health due to ART adherence which increased desire for sex resulting in an increase in pregnancy in some cases [41]. Studies also reported restored hope and a feeling of optimism about pregnancy among women living with HIV in South Africa and pregnant women receiving ART in Uganda, respectively [47, 51]. The sentiment that it would improve their immunity and therefore their chances of pregnancy was also noted in Senegal and South Africa [42, 45]. In one South African study among black women, participants directly stated that ART enabled them to meet the expectations of their partners and families to bear children stating, “this pill [a reference to ART] is important to give in-laws children” [45]. The same paper reported that healthcare workers either encouraged women to take ART to improve pregnancy chances while others stigmatized or discouraged women living with HIV from getting pregnant at all.

It was not always clear if the optimism about pregnancy was linked to participant perspectives that ART increased their biological ability to conceive (their fecundity) or whether it reflected perspectives about ART preventing vertical transmission or increasing their lifespan to be able to live long enough to care for their children. Despite these positive perceptions about ART and fecundity, some participants were still concerned about whether ART would allow them to live long enough to raise their children, and providers sometimes exacerbated these fears by only recommending one child [45].

In some studies, women reported that their provider advised them to “get healthy” or improve their CD4 count via ART before attempting pregnancy [44, 46]. In one study conducted in Kenya, women who were pregnant, recently postpartum or trying to conceive reported that this felt overly intrusive as this could delay their pregnancy. They felt that the clinic was restricting them and expecting them to ask for permission to become pregnant. They felt the tests done by the clinic to decide on their capability to become pregnant unnecessary [44]. In South Africa, a study which documented medical providers’ and policymakers’ perceptions, some participants stated that women should have a sufficiently high CD4 count and access to ART before getting pregnant, while others emphasized the importance of reproductive rights to decide on their own when they want to get pregnant [52].

### Stigma and Lack of Clear Guidance from Providers Discourage Women Living with HIV from Seeking Preconception Care

Although women living with HIV can safely conceive and give birth if they adhere to ART, studies reported that many women felt stigma from their provider, especially if they wanted a second or third child, and this discouraged them from seeking care. Some health care workers reported being open to discussions of fertility plans but data from patients suggest that providers can dissuade women living with HIV who would like to get pregnant, as documented in South Africa among black women [45], and in Botswana among women living with HIV and sexual and reproductive health/HIV providers [48]. Therefore, authors report that women often do not seek preconception counseling/care due to anticipated stigma from providers that having multiple children while living with HIV is risky [45]. Some providers voiced uneasiness about repeat pregnancies, suggesting that one pregnancy was acceptable but repeat pregnancies were concerning because of health risks for the mother and transmission risks for the partner and infant. When trying to communicate these concerns, providers sometimes used language that suggested to women living with HIV that they were discouraging pregnancy [45, 46, 48]. One study reported that social workers seem to be more person-centered around women’s desire to have children than medical providers [42] presenting a potential task-shifting or training opportunity.

### Lack of Awareness Among Both Providers and Patients About the Impact of HIV on Pregnancy, Highlights the Need for Provider Training

Most of the papers in this review (8 out of 14) mentioned a lack of understanding about how HIV and ART affect fecundity. This was true for both providers and patients. Several papers mentioned a notable gap in provider training around HIV and sexual and reproductive health and a call to increase community health worker training specifically [46, 47]. In Tanzania, a study among healthcare providers and men and women living with HIV found that some healthcare providers felt limited in their capacity to offer their clients living with HIV counseling on safe conception and pregnancy as they were not adequately trained [46]. Authors mentioned this is critical because as previously mentioned, patients reported that some providers advise them not to have children or not to have more than one child [45, 46, 48].

One study reported, “women living with HIV require information on the impact of HIV on pregnancy outcomes and vice versa” [45]. This was common across studies; several studies reported that women believed that pregnancy would weaken their immune system thereby worsening their overall health and not getting pregnant was best for their health [45, 49]. Current HIV services for women of reproductive age do not appear to proactively address issues of pregnancy and HIV progression, need to change ART regimens when considering or while pregnant, and post-partum recovery [45]. Studies reported that integration of HIV and sexual and reproductive health services and specific guidelines to support people living with HIV and their partners in planning pregnancies and managing their health is paramount [46, 52]. Another study advocated for including men in reproductive health and HIV educational efforts as they are often important decision makers and women may want their input and support [42].

In the only quantitative study included in this review, conducted in Ghana among healthcare providers, participants reported low knowledge about HIV and reproductive health in general [50]. Only about 10% of medical providers were aware of some reproductive options for women living with HIV. Some providers openly expressed their inability to give qualified and relevant advice to women living with HIV [50]. In a qualitative study with medical providers, they reported the importance of ART counseling to ensure that women who want to get pregnant adhere but in the same study, policy makers acknowledged a lack of clinical guidelines on how to discuss ART and fertility [52]. While there is a dearth of research on perceptions about HIV, ART, and fecundity, there was a clear overall trend that providers are ill-prepared to discuss how HIV and ART affect fecundity with their patients.

## Discussion

This systematic scoping review resulted in a scarcity of papers that reported perceptions about HIV or ART and fecundity in SSA. From the 12 included papers, there were beliefs that HIV, and one HIV prevention technology, cause infertility but perceptions around ART’s effect on fecundity were predominantly positive. The review also illustrated a lack of understanding of the relationship between HIV and fecundity among both people living with HIV and providers with several calls for additional provider training and better integration of HIV and reproductive health services.

Our search strategy identified a plethora of research on safe conception strategies and technologies for sero-different partners [4, 46, 53–57], a large body of work on prevention of vertical transmission thanks to ART [58–63], a good deal of work on fertility desires [64–67], and of course, the basis of this paper, clear research including systematic reviews across countries showing that HIV negatively impacts fecundity [2–4], and ART attenuates this effect [25–28]. Given that many of the research studies on HIV’s negative effect on fecundity and the protective effect of ART were published over a decade ago, we were surprised to find so few studies (and only one quantitative study) reporting on knowledge, attitudes, and perceptions of this phenomenon. The current state of the literature has been on more proximal concerns like how ART affects lifespan, preventing vertical transmission, and reducing stigma around people living with HIV having children at all. While we intentionally, developed a broad search to include concepts related to our primary research question (perceptions about HIV, ART, and fecundity), we also included adjacent topics (e.g., safe conception, fertility desires, etc.) with the idea that our research question could be embedded within the findings when we read the full text. It is also important to note that many of these studies took place before ART was recommended for everyone diagnosed with HIV in 2015. Therefore, awareness around HIV, ART, and fecundity would have likely improved since then and stigma around HIV has also changed. However, policy implementation often takes years to change following guidelines and community level perceptions and stigma can also take a long time to shift. Additionally, we note that the belief that “ART improves health” may not reflect beliefs about biological fecundity, the focus of this review, but overall health or a vague understanding of how it could improve health. This distinction is important because an understanding that ART could improve one’s fecundity or ability to get pregnant, may be a driver of ART adherence.

In sum, while the belief that contraceptives cause infertility is a well-documented barrier to contraceptive use, the belief that ART facilitates pregnancy could be a driver for ART adherence. To our knowledge, this has yet to be included in ART adherence education nor to be tested in ART adherence programs. Next steps from this work point to the importance of conducting qualitative research with men and women living with HIV to better understand their perceptions of this phenomenon and their influence on behaviors. Given that several studies reported a lack of training and perceived stigma around preconception counseling, research to better understand HIV providers’ knowledge and current practices is also warranted. Furthermore, given that knowledge, beliefs, and attitudes change over time, future research could develop a module for insertion within a nationally representative population-level survey and then measure changes in perceptions about HIV, ART, and fecundity. This approach would enable researchers to understand which factors are associated with these beliefs and how beliefs differ by geography.

Finally, the cultural importance of motherhood and childbearing in SSA cannot be underestimated. Given that several longitudinal studies show that HIV negatively impacts fecundity (e.g., time to pregnancy) and ART improves it, raising awareness among people living with HIV who would like to have children in the future, could be the difference between adhering to ART or not [68]. While we have a plethora of research on vertical transmission and preventing transmission among sero-discordant partners, people with HIV may not know that it impacts their fecundity. A particularly poignant time to raise awareness could be when or soon after adolescent girls and young women are diagnosed with HIV. Having the knowledge that their immediate actions, (regular ART adherence), could have long term benefits for their fecundity and not taking it could have negative impacts, could make a population level difference in ART adherence across SSA. Oftentimes, people are motivated to change behavior and improve their health for the sake of others, and in this case, their future children.

### Strengths and Limitations

This study has several strengths including our multi-disciplinary team from diverse geographies and backgrounds, inclusion of both French and English studies, and an applied approach that reports recommendations for future interventions and research. We also followed recommended frameworks and guidelines like the PRISMA guidelines for scoping and systematic reviews including updates to existing frameworks [68].

This study also has some limitations that should be taken into consideration while interpreting the results. Specifically, although we used a systematic, transparent, and replicable process to identify all relevant research from one authoritative and comprehensive biomedical and health sciences literature database, we did not search databases outside of PubMed or conduct a grey literature search. Limiting our search to PubMed may have led us to miss relevant studies indexed exclusively in other databases (e.g., Embase, PsycINFO, Web of Science, Scopus), particularly those published in non-biomedical journals. To mitigate this risk, we used a sensitive MeSH- and keyword-based strategy, screened reference lists of all included studies, and solicited input from our multidisciplinary author team to identify additional eligible papers. Additionally, due to the scoping nature of our research questions, we did not conduct a quality assessment of each paper included in the review. Furthermore, we did not include research from before the year 2000 and papers may have included findings from the beginning of the HIV epidemic. Additionally, our search is limited to SSA, so findings may not be relevant to other regions. However, focusing on this region is also a strength given the high prevalence of women of reproductive age living with HIV in this continent. Furthermore, findings reflect heterogeneous and often small samples. Finally, given the scarcity of literature, we could not make meaningful comparisons across time points (e.g., changes in beliefs before and after universal ART scale up or across anglophone and francophone contexts).

In conclusion, the results of this review provide important conceptual groundwork for programs that aim to raise awareness among women, men, their providers, and social networks about the relationship between HIV, ART, and fecundity to both increase ART adherence and help people living with HIV build their families. While our findings do not point to specific intervention guidance, perhaps a light touch, low-cost intervention could be warranted either using patient to provider education, technology, or mass communication. To our knowledge, this approach has not been tested. Future research is needed to understand if addressing this gap in understanding could improve ART adherence and help couples living with HIV build their families.

## Supplementary Information

Below is the link to the electronic supplementary material.

Supplementary material 1 (DOCX 14.2 kb)

## Data Availability

Data sharing not applicable to this article as no datasets were generated or analyzed during the current study.

## Abbreviations

HIV: Human immunodeficiency virus
ART: Antiretroviral therapy
PRISMA: Preferred Reporting Items for Systematic Reviews and Meta-Analyses
