# Supplementary material for: Perceptions About the Relationship Between HIV, Antiretroviral Therapy (ART), and Fecundity in Sub-Saharan Africa: Findings from a Systematic Scoping Review
Source: AIDS Behav. 2026 Apr 11;30(10):3172–86. doi: 10.1007/s10461-026-05111-8 (PMC13162283; doi:10.1007/s10461-026-05111-8)
Supplement: Supplementary file 1 [file 10461_2026_5111_MOESM1_ESM.docx]

**Supplementary Material**

Supplemental File 1 Excluded Studies on Full Text Screening

1. Bankole et al. (2014): Does knowledge about antiretroviral therapy and mother-to-child transmission affect the relationships between HIV status and fertility preferences and contraceptive use? New evidence from Nigeria and Zambia.

*Reason for exclusion: No mention of understanding of the relationship between HIV, ART and fertility.*

2. Beyeza-Kashesya et al. (2010): My partner wants a child: A cross-sectional study of the determinants of the desire for children among mutually disclosed sero-discordant couples receiving care in Uganda.

*Reason for exclusion: No mention of understanding of the relationship between HIV, ART and fertility.*

3. Garver et al. (2015): Changing Childbearing Norms During an Era of ART Expansion in Malawi, 2009 to 2015.

*Reason for exclusion: No mention of understanding of the relationship between HIV, ART and fertility.*

4. Gutin et al (2021): Relationship, partner factors and stigma are associated with safer conception information, motivation, and behavioral skills among women living with HIV in Botswana.

*Reason for exclusion: No mention of understanding of the relationship between HIV, ART and fertility.*

5. Iliyasu et al. (2019): Health workers' knowledge of safer conception and attitudes toward reproductive rights of HIV-infected couples in Kano, Nigeria.

*Reason for exclusion*: *No mention of understanding of the relationship between HIV, ART and fertility.*

6. Kimemia et al. (2019): Knowledge, attitude and practice of child adoption among infertile female patients in Sokoto north-west Nigeria

*Reason for exclusion*: *No mention of understanding of the relationship between HIV, ART and fertility.*

7. Kisakye et al. 2010: Pregnancy decisions among HIV-positive pregnant women in Mulago Hospital, Uganda.

*Reason for exclusion*: *No mention of understanding of the relationship between HIV, ART and fertility*.

8. London et al. (2008): 'Even if you're positive, you still have rights because you are a person': human rights and the reproductive choice of HIV-positive persons.

*Reason for exclusion: No mention of understanding of the relationship between HIV, ART and fertility.*

9. MacGregor et al. (2011): Framing rights and responsibilities: accounts of women with a history of AIDS activism.

*Reason for exclusion: No mention of understanding of the relationship between HIV, ART and fertility.*

10. Magadi & Agwanda (2010): Investigating the association between HIV/AIDS and recent fertility patterns in Kenya

*Reason for exclusion: No mention of understanding of the relationship between HIV, ART and fertility.*

11. Moodley et al. (2014): Health care provider perspectives on pregnancy and parenting in HIV-positive individuals in South Africa.

*Reason for exclusion: No mention of understanding of the relationship between HIV, ART and fertility.*

12. Moore et al. (2015): Attitudes of women and men living with HIV and their healthcare providers towards pregnancy and abortion by HIV-positive women in Nigeria and Zambia.

*Reason for exclusion: No mention of understanding of the relationship between HIV, ART and fertility.*

13. Moore et al. (2014): Bucking social norms: Examining anomalous fertility aspirations in the face of HIV in Lusaka, Zambia.

*Reason for exclusion: No mention of understanding of the relationship between HIV, ART and fertility.*

14. Ngure et al. (2014): My intention was a child but I was very afraid: fertility intentions and HIV risk perceptions among HIV-serodiscordant couples experiencing pregnancy in Kenya.

*Reason for exclusion: No mention of understanding of the relationship between HIV, ART and fertility.*

15. Ngure et al. (2014): Delivering safer conception services to HIV serodiscordant couples in Kenya: perspectives from healthcare providers and HIV serodiscordant couples.

*Reason for exclusion: No mention of understanding of the relationship between HIV, ART and fertility.*

16. Mujumdar et al. (2018): Reproduction and Fertility Beliefs, Perceptions, and Attitudes in People Living with HIV.

*Reason for exclusion: No mention of understanding of the relationship between HIV, ART and fertility.*

17. Leyva-Moral et al. (2018): Reproductive decision-making in women living with human immunodeficiency virus: A systematic review.

*Reason for exclusion: No mention of understanding of the relationship between HIV, ART and fertility.*

18. Haddad et al. (2016): Influences in fertility decisions among HIV-infected individuals in Lilongwe, Malawi: A qualitative study.

*Reason for exclusion: No mention of understanding of the relationship between HIV, ART and fertility.*

19. Brown JM, Gitome S, Mataveke B, et al. Preventing HIV and achieving pregnancy among HIV sero-different couples: Pilot study of a safer conception intervention in Zimbabwe. Pai M, ed. PLOS Glob Public Health. 2023;3(2):e0000796. doi:10.1371/journal.pgph.0000796

*Reason for exclusion: No mention of understanding of the relationship between HIV, ART and fertility.*

20. Gitome S, Musara P, Chitukuta M, et al. (2024). “First was to sit down and bring our minds together”. A qualitative study on safer conception decision-making among HIV sero-different couples in Zimbabwe. Sex Reprod Health Matters. 2024;32(1):2366587. doi:10.1080/26410397.2024.2366587

*Reason for exclusion: No mention of understanding of the relationship between HIV, ART and fertility.*
